# ErbB4 in the brain: Focus on high grade glioma

**DOI:** 10.3389/fonc.2022.983514

**Published:** 2022-08-31

**Authors:** Jamie-Lee Pitcher, Naomi Alexander, Panimaya Jeffreena Miranda, Terrance G. Johns

**Affiliations:** ^1^ Oncogenic Signalling Laboratory, Telethon Kids Institute, Nedlands, WA, Australia; ^2^ School of Biomedical Sciences, University of Western Australia, Crawley, WA, Australia; ^3^ Division of Paediatrics/Centre for Child Health Research, University of Western Australia, Crawley, WA, Australia

**Keywords:** ErbB4, EGFR, receptor tyrosine kinase, high grade glioma, diffuse midline glioma

## Abstract

The epidermal growth factor receptor (EGFR) family of receptor tyrosine kinases (RTKs) consists of EGFR, ErbB2, ErbB3, and ErbB4. These receptors play key roles in cell proliferation, angiogenesis, cell migration, and in some cases, tumor promotion. ErbB4 is a unique member of the EGFR family, implicated not only in pro-tumorigenic mechanisms, such as cell proliferation and migration, but also in anti-tumorigenic activities, including cell differentiation and apoptosis. ErbB4 is differentially expressed in a wide variety of tissues, and interestingly, as different isoforms that result in vastly different signalling outcomes. Most studies have either ignored the presence of these isoforms or used overexpression models that may mask the true function of ErbB4. ErbB4 is widely expressed throughout the body with significant expression in skeletal tissue, mammary glands, heart, and brain. Knockout models have demonstrated embryonic lethality due to disrupted heart and brain development. Despite high expression in the brain and a critical role in brain development, remarkably little is known about the potential signalling activity of ErbB4 in brain cancer.This review focuses on the unique biology of ErbB4 in the brain, and in particular, highlights brain cancer research findings. We end the review with a focus on high grade gliomas, primarily glioblastoma, a disease that has been shown to involve EGFR and its mutant forms. The role of the different ErbB4 isotypes in high grade gliomas is still unclear and future research will hopefully shed some light on this question.

## Overview of ErbB4 biology

The epidermal growth factor receptor (EGFR) family of receptor tyrosine kinases (RTKs), also known as the ErbB family of receptors, comprises four members: EGFR/ErbB1/HER1; ErbB2/Neu/HER2; ErbB3/HER3; and ErbB4/HER4 (1). These receptors play key roles in cellular proliferation, differentiation, migration, angiogenesis, and apoptosis (1). Therefore, unsurprisingly, aberrant signalling through these receptors has been implicated in the pathogenesis of numerous tumor types.

**Figure 1:**
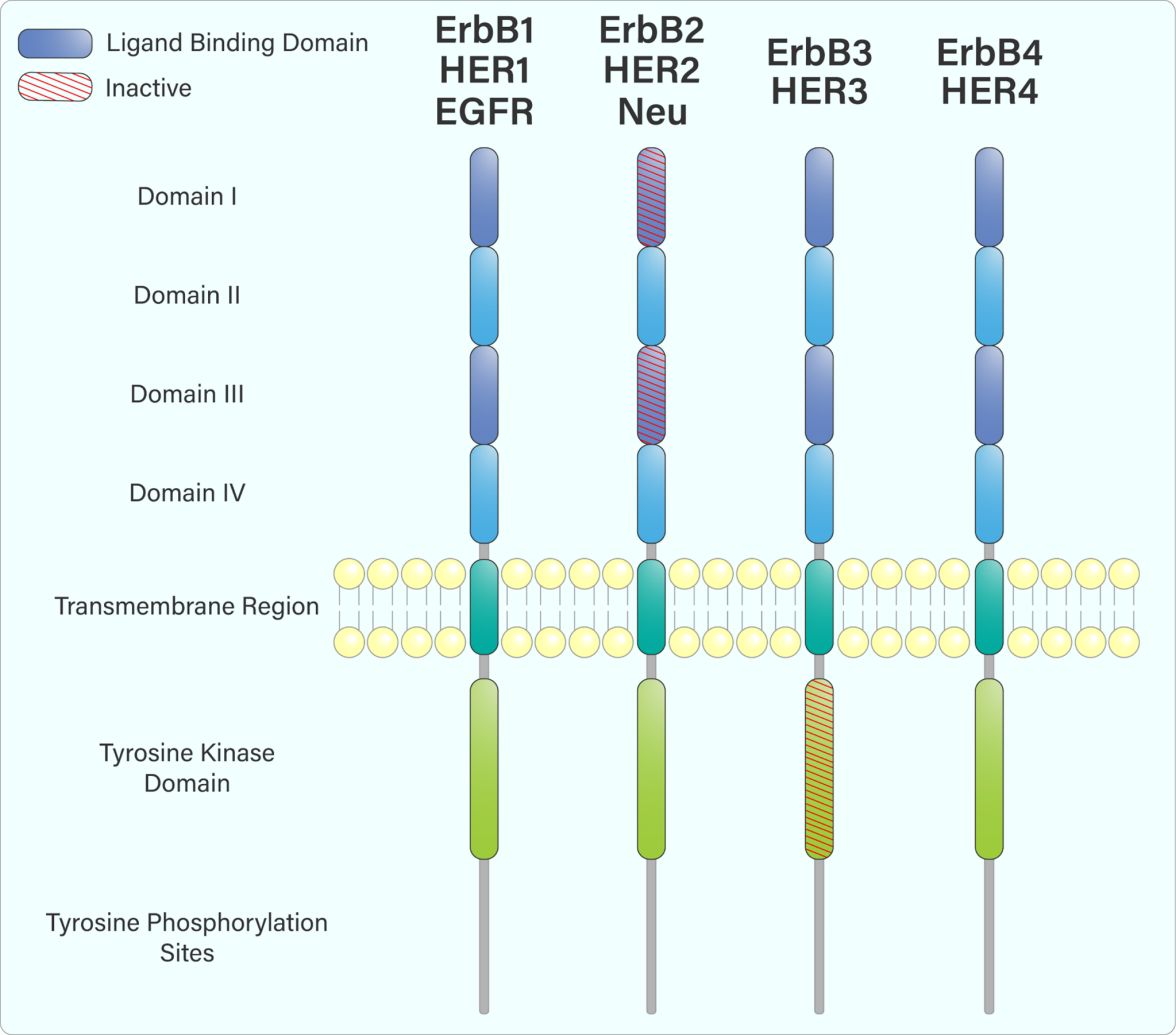
The domain arrangement of the ErbB receptor family. The extracellular region is composed of 4 domains: I, II, III & IV. Domains I and III (purple) form an extracellular ligand-binding domain that facilitates ligand attachment at the cell surface. ErbB2, while containing a ligand-binding domain, does not bind any ligands. Upon ligand engagement, the ErbB family members hetero- or homodimerize with each other, leading to trans-autophosphorylation of tyrosine residues within their cytoplasmic tails through their intracellular tyrosine kinase domain. ErbB3 does not have an active tyrosine kinase domain and must heterodimerize with another ErbB receptor to be phosphorylated.

The ErbB receptors share similar domain organization, including an extracellular ligand-binding domain, a single membrane-spanning region, an intracellular tyrosine kinase domain, and a C-terminal region containing various tyrosine residues (**Figure 1**) (2). Following ligand engagement of these receptors at the cell surface, hetero- or homodimerization occurs, leading to trans-autophosphorylation of tyrosine residues within their cytoplasmic tails. This creates binding or docking sites for adaptor and signalling proteins containing SH2 and PTB domains, such as Grb2, Shc, and PI3-Kinase (PI3-K) (3, 4).

**Figure 2:**
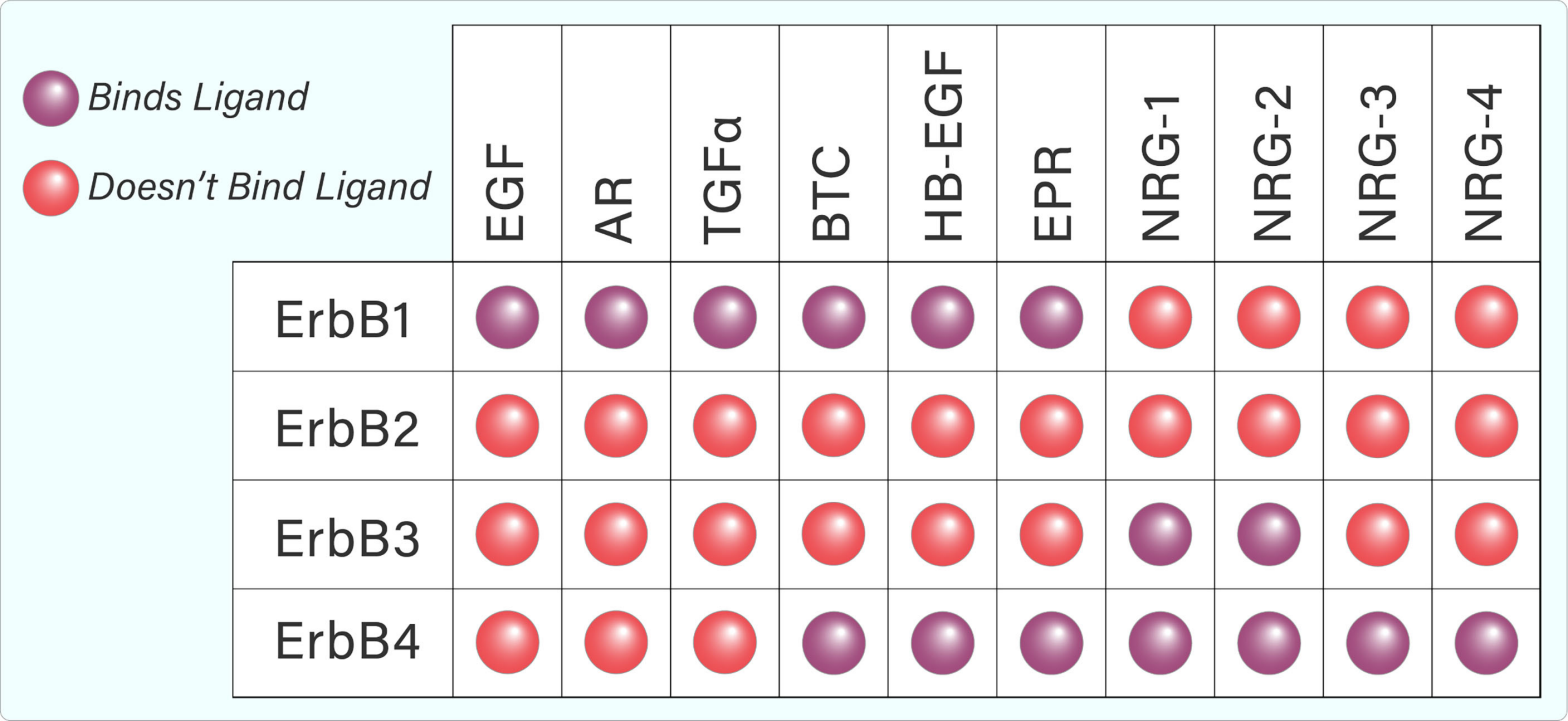
Tabular representation of ErbB ligand binding. ErbB1, 3, and 4 are capable of binding several ligands while ErbB2 does not appear to bind any known ligands.

Several ligands are capable of binding to and activating the ErbB family. Each ErbB-binding ligand contains an EGF-like domain, which helps to confer binding specificity, and allows the ligand to preferentially or specifically bind to a particular ErbB family member (1). Whilst ErbB1, 3, and 4 bind several ligands, ErbB2 does not appear to bind any known ligands (**Figure 2**) (5). However, ErbB2 dimerizes with other ErbB members, which helps to stabilize ligand interactions. Furthermore, it appears that ErbB2 is the preferred dimerization partner for other ErbB family members (5).

ErbB4 was cloned in 1993 and shows similar domain organization to the other, previously identified, members of the ErbB family (6, 7). Specifically, the ecto- and cytoplasmic domains are separated by a single transmembrane domain (8). The extracellular domain contains two cysteine-rich regions and displays high levels of similarity to the extracellular domain of ErbB3 (7, 8). The cytoplasmic domain is more homologous to the cytoplasmic domains of EGFR and ErbB2 and is composed of a juxtamembrane (JM) region, a tyrosine kinase domain, and a carboxy terminal tail (7, 8).

## ErbB4 domain structure

**Figure 3:**
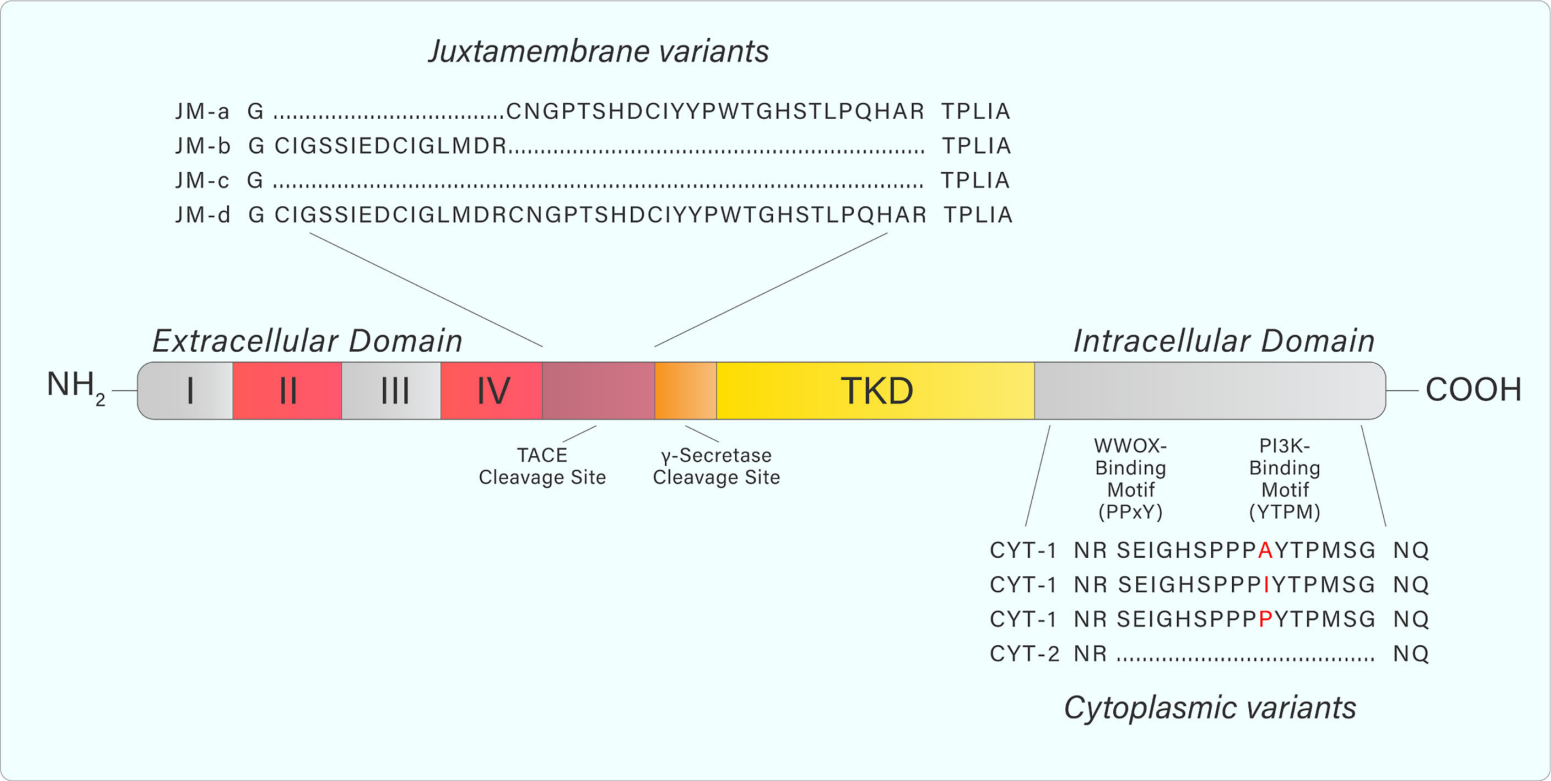
Adapted from Donoghue et al. Graphic representation of ERBB4 juxtamembrane (JM-a, b, c, d) and cytoplasmic (CYT-1, 2) variant sequences. JM-a- and JM-d variants contain tumor necrosis factor converting enzyme (TACE) and γ-secretase cleavage sites causing release of the extracellular domain and subsequent release of a soluble intracellular fragment. Variants containing JM-b and JM-c do not undergo cleavage and remain as a full-length receptor. CYT-1-containing variants include a 16-amino acid sequence that contains WW domain-containing oxidoreductase (WWOX)- and PI3K-binding motifs. These binding motifs are absent from CYT-2-containing variants. Amino acid sequences were generated from the NCBI Nucleotide database.

ErbB4 structure and signalling is complicated by the existence of different splice variants (9). Specifically, exon splicing occurs within the *ERBB4* JM domain to produce the splice variants JM-a, JM-b, JM-c, and JM-d (**Figure 3**) (9–11). A cleavage site for the tumor necrosis factor-alpha converting enzyme (TACE) is encoded within exon 16 of *ERBB4* (9). The JM-a and JM-d variants both contain exon 16, and therefore, contain the cleavage site (9). In contrast, the ErbB4 JM-b and JM-c variants do not contain exon 16 (9). Additional splicing results in alterations to the cytoplasmic domain (towards the C-terminal tail), producing the CYT-1 and CYT-2 variants by the inclusion or exclusion of exon 26 (9). This results in the respective presence or absence of a 16-amino acid sequence (Ser1046 through Gly1061) (11–13).

**Figure 4:**
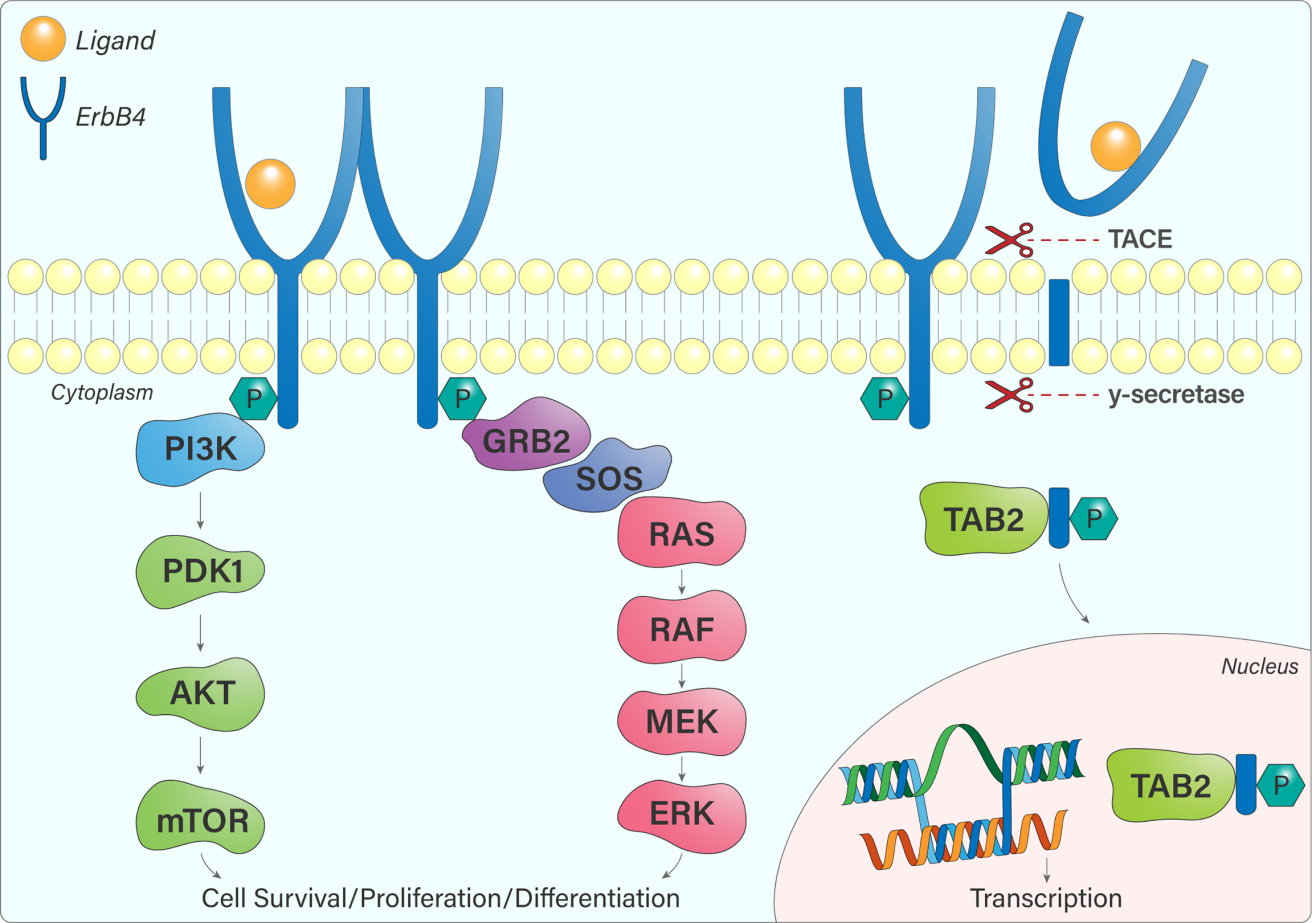
ErbB4 signalling pathways. Once ErbB4 is phosphorylated, signalling cascades become activated, notably the PI3K-AKT and Ras-MAPK pathways that influence cell survival, proliferation, and differentiation. JM-a or JM-d-containing ErbB4 variants are cleaved by TACE and γ-secretase, resulting in the release of a soluble intracellular fragment that forms a complex with TAB2 and undergoes nuclear translocation. In the nucleus, ErbB4 regulates gene transcription.

The different ErbB4 isoforms allow for various signalling outcomes. Subsequent to ligand engagement, cleavage of protease-sensitive ErbB4 isoforms (JM-a, JM-d) within the JM domain (between His-651 and Ser-652) by TACE releases the extracellular domain and leaves a membrane-bound intracellular domain (ICD), known as m80. This triggers a secondary cleavage event by γ-secretase, releasing an intracellular domain fragment (s80), which can then translocate to the nucleus and interact with transcriptional regulators (14, 15). Moreover, ErbB4 contains a Nuclear Localization Sequence, providing further evidence for its role as a transcriptional regulator, which is unique within the ErbB receptor family (16, 17). The 16 additional amino acids present within the CYT-1 isoform contain docking sites for SH2 domains and WW-domain-containing proteins (15). This allows, for example, CYT-1 to signal through the PI3-Kinase pathway, whereas CYT-2 lacks this ability (**Figure 4**) (12).

## Ligand binding

The ErbB family is activated by ligands that share an EGF-like motif of 45–55 amino acids (18). Binding specificity is conferred by loops that form within this motif due to covalent interactions between six cysteine residues (18). The ligands can be separated into three groups – those that specifically bind EGFR, those that bind ErbB4 and EGFR (epiregulin, betacellulin, heparin-binding EGF) and the neuregulins (NRG), which specifically bind ErbB4 and ErbB3 (**Figure 2**) (19). Neuregulin (denoted as Heregulin in humans) belongs to a family of EGF-like polypeptides that are widely expressed. They are encoded by at least six genes (*NRG-1*–*6*), each of which have multiple splice forms (19). NRG-1 was the first member of the family to be discovered and is the best-characterized (19, 20). The neuregulins can again be divided into two groups, depending on their ability to bind ErbB3 and ErbB4 (NRG-1, NRG-2), or ErbB4 only (NRG-3, NRG-4). Early studies found that NRG-1 is a preferred ligand for ErbB4 (7, 21). Binding analyses showed that ErbB4/ErbB2 heterodimers have greatly increased affinity for heregulin, well surpassing that of ErbB3/ErbB4 heterodimers or ErbB4 homodimers (22). The ability of ErbB4 to bind a variety of ligands from both the EGF-like and NRG families contributes to its uniqueness in the ErbB family and has the potential to diversify signalling outcomes even further.

## Role of ErbB4 in brain development

An RNA analysis study demonstrated that ErbB4 is highly expressed in brain, heart, and skeletal muscle (7). A subsequent study showed that ErbB4 mRNA is expressed in the hindbrain, mid-brain, and ventral forebrain at E9.5 in developing mouse embryos and the protein is detectable in brain tissue of both embryonic and adult mice (23).

Studies addressing specific variant expression in mice have shown that the mouse kidney solely expresses the JM-a variant and the heart solely expresses the JM-b variant, whilst the cerebellum expresses both variants (6). With regards to the cytoplasmic domain variants, CYT-1 is mainly expressed in mouse heart, mammary gland, and skin, while CYT-2 is the main variant in kidney and neural tissues (12). This tissue-specific expression of ErbB4 juxtamembrane and cytoplasmic variants further complicates signalling outcomes.

An essential role for ErbB4 in the central nervous system (CNS) has been demonstrated in mice by the creation of ErbB4 null mutants, which are terminal at E10.5 due to aborted development of myocardial ventricular trabeculae and disruptions to innervation of the hindbrain (23). In the CNS, ErbB4 is the primary receptor for NRG-1 (24, 25). This NRG-1-ErbB pathway is crucial for the proliferation, differentiation, and survival of glial cells, as well as the development of Schwann cells in the peripheral nervous system (26). This pathway also has an important role in inducing receptors for neurotransmitters such as acetylcholine, gamma-aminobutyric acid (GABA), and N-Methyl-D-aspartic acid, as well as regulating dendritogenesis in the cerebellum (27–29). Studies supporting these crucial roles have reported expression of both ErbB4 and NRG-1–4 in the brain (7, 19).

The different NRG isoforms are the products of alternative splicing of a single gene, which is regulated by neuronal activity (30, 31). There are more than 30 isoforms of NRG-1 alone, and these isoforms are categorized into six groups (32). In rats, mRNA analysis has shown that at different ages, each group has a distinctive expression pattern in the brain (30). This is consistent with the previously established role of NRG-1 in neural development. However, there are significantly fewer studies focusing on the NRG-2–4 isoforms. NRG-1 is highly expressed in the embryonic and fetal brain and expression declines during postnatal development. In contrast, NRG-2 levels significantly increase soon after birth (33, 34). This indicates that NRG-1 is vital in prenatal development while NRG-2 has an essential role in the postnatal and adult brain. NRG-2, -3, and -4 stimulate ErbB4 tyrosine phosphorylation (35). Furthermore, NRG-2 and -3 stimulation couples ErbB4 activation to biological responses and NRG-3 is a critical mediator in cortical inhibitory circuit assembly (35, 36). Until recently, NRG-4 was not demonstrated to have a role in neural development (37). However, it has since been shown to have an important role in the regulation of dendritic arborization in the developing cerebral cortex (37). Additionally, NRGs and ErbB4 are essential for synapse formation, interneuron migration, and axon and dendrite development, all of which are necessary for the GABAergic circuitry assembly that controls neural activity in the cerebral cortex (25).

As defective heart development is the reason for lethality in ErbB4 KO mice, one group expressed ErbB4 under a cardiac-specific myosin promoter in these mice, allowing them to reach adulthood (38). Consequently, the potential developmental role of ErbB4 in the brain and other tissues could be studied. Authors observed aberrant cranial neural crest cell migration, abnormal cranial nerve architecture, and significantly more interneurons in the cerebellum of the heart-rescued ErbB4 KO mice (38).

In the developing rat brain, Yau and colleagues analyzed ErbB4 immunoreactivity and showed that ErbB4 is preferentially expressed in interneurons migrating tangentially from the ventral to the dorsal telencephalon (25). ErbB4 mRNA was also detected at high levels in the cerebral cortex from prenatal day 18–20, peaking at postnatal day 0 (day of birth), hinting at the regulatory role of ErbB4 on neural development. Comparable results were reported by Fox and Kornblum, who found high ErbB4 expression in the germinal zones surrounding the lateral ventricles of the mouse forebrain only from prenatal day 17 through to postnatal day 1 (39).

Intriguingly, the high expression of the JM-a isoform in the brain (6) suggests ErbB4 cleavage and nuclear activity could play an important role in this tissue (40). The ability of ErbB4 to translocate into the nucleus and alter transcription has been associated with diverse outcomes, including poor prognostic outcomes in breast cancer (41). In the brain, ErbB4 signalling (specifically, the nuclear translocation of the ErbB4 ICD) is thought to be critical for the timing of astrogenesis in the developing brain (40). Interaction of the ErbB4 ICD with the signalling protein TAB2 and the corepressor N-CoR leads to nuclear translocation of a complex that can repress glial gene transcription and prevent neural precursor cells from differentiating into astrocytes (40).

## Role of ErbB4 in brain injury, neurodegenerative diseases, and psychological disorders

ErbB4 has been implicated in neuroprotection from brain ischemia, *via* a signalling mechanism involving NRG-1β activation that protects against oxygen-glucose deprivation-induced neuronal death (42). Experiments in rodent models have also linked ErbB4 signalling to neuronal protection after brain injury and hemorrhage (43–45). Multiple studies have found ErbB4 to be critical for the assembly of the GABAergic (inhibitory) system (19). Alterations in this circuitry have been associated with the development of neurodevelopmental diseases, neurological and psychological disorders, and neuronal plasticity (46, 47). Furthermore, ErbB4 is expressed at the presynaptic terminals of GABAergic interneurons (48, 49), with detailed analysis narrowing this down to parvalbumin-expressing interneurons (50). Although an extensive coverage of the literature in this field is beyond the scope of this review, we will briefly summarize some of the research addressing the role of ErbB4 in schizophrenia, Alzheimer’s disease (AD), and Parkinson’s disease (PD).

Several studies have identified intronic variants of ErbB4 that are associated with schizophrenia, AD, and PD (51–54). The *ERBB4* and *NRG-1* genes have been implicated as risk genes for the development of schizophrenia. Indeed, the Neuregulin-ErbB4 signalling pathway plays a critical role in the development of inhibitory circuits in the mammalian cortex (50, 55). Mice hypomorphic for *NRG-1* or *ERBB4* demonstrate similar behavioral abnormalities and anti-psychotic drug treatment of the *NRG-1* hypomorphs is able to partially reverse the behavioral phenotype (56).

Fifteen ErbB4 sequence variants have been associated with schizophrenia. Moreover, one of these variants is associated with an increased risk of schizophrenia in people carrying the Icelandic NRG-1 risk haplotype. Two ErbB4 alleles are significantly overexpressed in Ashkenazi schizophrenia patients, compared with matched controls (57). Other studies have identified additional genetic variants of the ErbB4 gene associated with this disease (58–60). Neuregulin signalling through ErbB4 selectively decreases fast synaptic GABA_A_ currents on hippocampal interneurons which may contribute to the pathophysiology of epilepsy and neuropsychiatric disorders (61).

A 2010 study found significantly higher ErbB4 immunoreactivity in apoptotic hippocampal pyramidal neurons in AD patients, compared with healthy controls (62). These results, the authors argue, reflect a link between ErbB4 up-regulation and the progression of AD pathology (62). In a subsequent study, the same group found increased ErbB4 expression in neurons from the cortico-medial nucleus amygdala, human basal forebrain, and superior frontal gyrus of patients with AD (63). Additionally, ErbB4 is found at high levels surrounding the neuritic plaques that are characteristic of AD and potential allelic variants of ErbB4 have been associated with AD (64). Similarly, there is elevated ErbB4 expression in midbrain tissue sections of PD patients, compared with healthy controls.

## The role of ErbB4 in cancer – oncogene, tumor suppressor, or both? the influence of isoforms

The different ErbB4 isoforms and their variable tissue expression may help to explain seemingly opposing signalling outcomes. Non-cleavable isoforms may dimerize with other ErbB members and recruit signalling proteins to the plasma membrane (65–67). Alternatively, proteolytic cleavage may result in translocation of the 80kDa intracellular domain to the nucleus, where it can participate as a transcriptional regulator (**Figure 3**) (14, 16, 65).

The ErbB4 isoform JM-a/CYT-2 is capable of oncogenic activity as a result of autoactivation, independent of the ligand binding that is required by other isoforms (68, 69). This ligand-independent autophosphorylation can increase cell proliferation and decrease apoptosis (52). Furthermore, the JM-a/CYT-2 isoform is known to promote survival and proliferation of breast cancer cells (68, 70). Specifically, overexpression of the JM-a/CYT-2 isoform combination promotes the proliferation of breast cancer cells, even in the absence of ligand (68). Conversely, expression of CYT-1 in breast cancer cell lines has tumor-suppressor effects by producing ligand-dependent growth inhibition and differentiation (71, 72). Results of such studies indicate isoform-specific roles of ErbB4 in cancer.

Interestingly, cytosolic localization of ErbB4 has been associated with better breast cancer prognosis, whilst nuclear localization is associated with worse prognosis (41). Real-time reverse transcription-PCR (RT-PCR) analysis of breast cancer samples determined that expression of the cleavable JM-a isoform is associated with estrogen receptor-α expression and a high histologic grade of differentiation (73). ErbB4 nuclear immunoreactivity is also associated with poor patient survival, compared with women whose cancer cells had membranous ErbB4 staining (73).

Mutations targeting the ErbB4 gene have been functionally characterized in some non-brain cancers, like non-small cell lung cancer (74). Kurppa and colleagues identified four specific mutations, located in the dimerization interface of the extracellular domain and in the tyrosine kinase domain (74). These mutations were functionally analyzed and found to increase basal and ligand-induced ErbB4 phosphorylation (74).

Similarly, novel ErbB4 mutations are present in 19% of melanoma patients and seven missense mutations in *ERBB4* have been found to increase transformation ability and kinase activity (75). ErbB4 gain-of-function mutations are believed to be used by melanoma cells to activate the PI3K pathway (76). Furthermore, mutant ErbB4 in melanoma cells is associated with increased cell growth and this growth is reduced when these cells are treated with the ErbB inhibitor lapatinib (75).Silencing endogenous ErbB4 in ErbB4 mutant-expressing melanoma cells leads to an arrest in AKT phosphorylation and cellular proliferation (75).

However, not all ErbB4 mutations are linked to cancer. For example, a recent study by Jones and colleagues analyzed data from the Cancer Cell Line Encyclopedia (CCLE) and the Cancer Genome Atlas (TCGA), focusing on copy number mutations in ErbB4 (77). The study found that a region in Intron 1 of the *ERBB4* gene was deleted in 69.1% of tumor samples harboring *ERBB4* copy number loss (77). However, the same deletion was found at a similar frequency in matched normal tissue samples from these glioblastoma patients and in the general population (77).

## The lack of isoform-specific and cleavage-specific reagents

Whilst ErbB4 is activated by a specific subset of ligands, once ligand-bound, it can homodimerize with other ErbB4 receptor molecules or heterodimerize with other ErbB family members. This complicates the study of ErbB4-induced signalling events (13). Compounding this, the lack of ErbB4-specific reagents can make it challenging to study its biological role. For example, the assessment of ErbB4 phosphorylation status can be difficult due to the lack of specific antibodies that are able to detect phosphorylation of specific tyrosine residues. A report from Gallo and colleagues found that an antibody directed against Tyr 1056, found within the ErbB4 CYT1 isoform but not the CYT2 isoform, produced a signal in mutant ErbB4 cells lacking this site (31). The authors showed that it not only cross-reacted with other ErbB4 phosphorylation sites, but also with phosphorylated EGFR (55). This highlights the importance of using immunoprecipitation in certain cases to verify the identity of phosphorylated ErbB4.

The existence of different ErbB4 isoforms also makes it inherently complicated to study the biological role of this receptor. It has been demonstrated in many publications that different ErbB4 isoforms produce varying signalling and biological outcomes. Furthermore, the generation of the ErbB4 intracellular domain (4ICD) can result in different subcellular localizations. Although there are currently no antibodies that specifically detect the different ErbB4 isoforms, antibodies have been developed that recognize the N-terminal or C-terminal of the receptor. A study by Tovey and colleagues compared antibody detection of ErbB4 in estrogen receptor-positive breast cancer patients (78). They used two different antibodies: the HFR1 antibody, which recognizes both the intact receptor and cleaved ICD, and the H4.77.16 antibody, which recognizes the extracellular domain of ErbB4, and thus only stains the full length receptor (78). The authors reported that patients demonstrating nuclear staining with the H4.77.16 antibody only had poorer survival (78). No such survival correlation was observed with the HFR1 antibody, suggesting that HFR1 may select for cytoplasmic and nuclear ErbB4 ICDs whilst H4.77.16 selects for membranous ErbB4 (79). These findings suggest that ErbB4 may be recycled in the cytoplasm or nucleus, however, further study is required for confirmation (80).

Due to the well-documented roles of EGFR and ErbB2 as oncogenes, much research has been directed at developing specific inhibitors against these family members, such as Gefitinib (EGFR inhibitor) and CP-724714 (ErbB2 inhibitor) (81, 82). Some of these have shown promise in the clinic, including the EGFR inhibitor Afatinib as a treatment for advanced non-small-cell lung cancer and the ErbB2 inhibitor Lapatinib in the treatment of advanced breast cancer (83, 84).

To date, there are no specific ErbB4 inhibitors. This makes it challenging to study the role of endogenous ErbB4. Many studies have used overexpression models to study the biological role of ErbB4. However, overexpression models, while helpful, have limitations and may not be truly representative of normal cellular function or the role of endogenous ErbB4 (85). Furthermore, many studies looking at expression do not take into account the different isoforms (85). In addition, the use of different ErbB4 antibodies can generate conflicting results (79).

## The role of ErbB4 in brain cancers, including high-grade glioma

**Figure 5:**
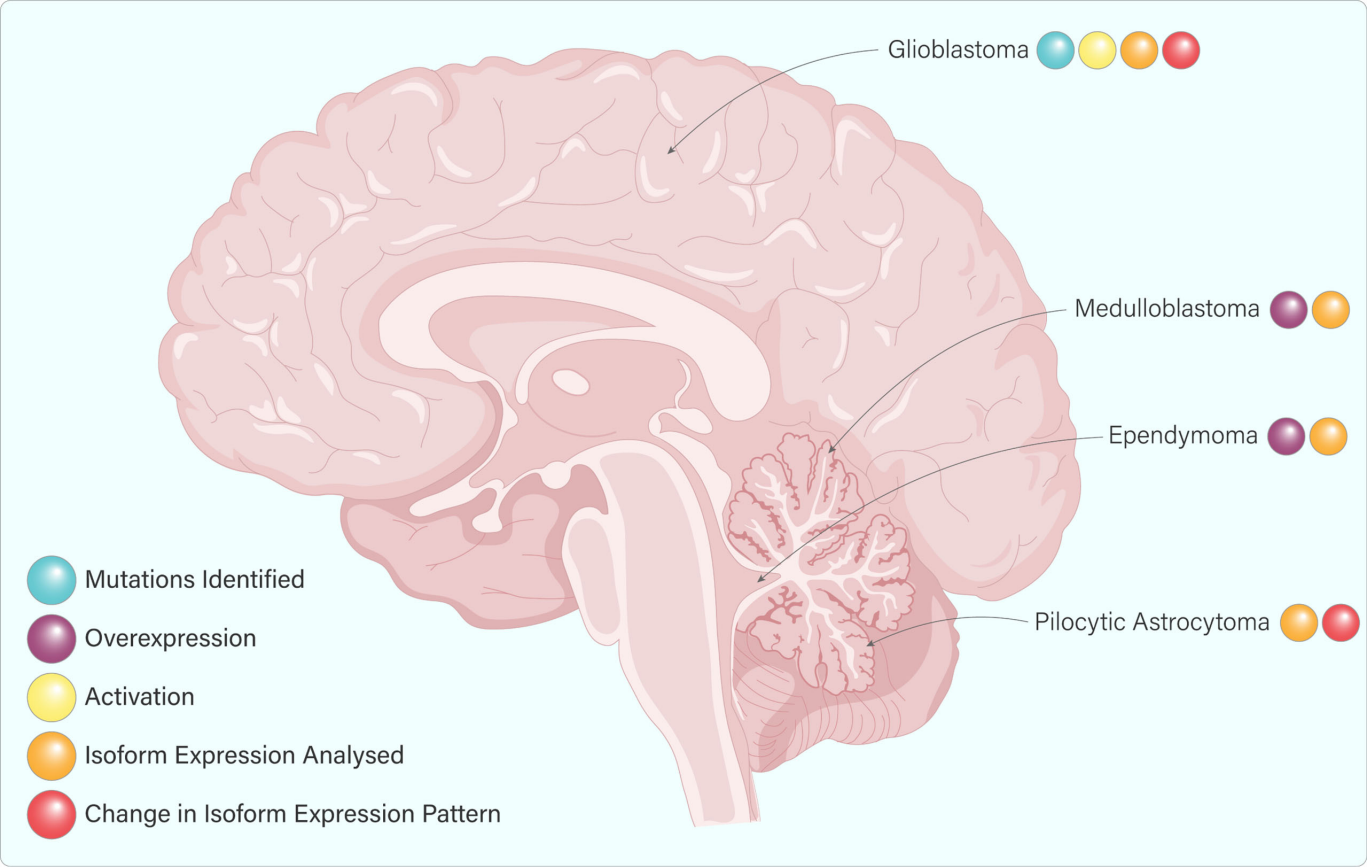
ErbB4 expression and activation in various brain cancers. ErbB4 activation and mutations have only been identified in Glioblastoma. ErbB4 overexpression is observed in medulloblastoma and ependymoma. Isoform expression has been analyzed in all four cancers displayed however only glioblastoma and pilocytic astrocytoma showed a change in the isoform expression pattern.

Whilst there is a wealth of literature on the potential role of ErbB4 in breast cancer, melanoma, and lung cancer, relatively little is known about its potential role in brain cancers. This is surprising, given its high expression in this tissue and the essential role it plays in brain growth and development. **Figure 5** highlights some of the available data on ErbB4 expression and/or activation in various brain cancers.

ErbB4 expression and activity is ubiquitous during brain development. As would be expected, dysfunctions of ErbB4 isoforms have been implicated in different pathologies, including malignancies. Overexpression of ErbB4 has been identified in medulloblastomas, pilocytic astrocytomas, ependymomas, and glioblastomas (GBMs) (**Figure 5**) (86–89). In most benign meningiomas, however, ErbB4 is underexpressed and co-expression of ErbB4 with ErbB2 has been associated with poor prognosis in medulloblastoma and with increased proliferation index in ependymomas (86–88, 90).

Medulloblastomas, characterized as Grade IV lesions and ranked as the most common solid tumors in childhood, arise from abnormal development of the cerebellum (91). Overexpression of ErbB4 in conjunction with ErbB2 correlates with poor prognosis in medulloblastoma patients (87) (92). Recent analysis of the relative RNA levels of ErbB4 isoforms has shed some light on the differential expression patterns present within medulloblastoma.

A paper by Zeng and colleagues analyzed the relative levels of the different JM and CYT isoforms of ERBB4 in two pediatric brain tumor types: highly malignant medulloblastoma and the relatively benign pilocytic astrocytoma (86). They found different ratios of expression of the JM isoforms, with predominantly more of the cleavable JM-a isoform than the non-cleavable JM-b isoform in medulloblastoma and pilocytic astrocytoma, whereas the opposite pattern occurred in normal brain. Furthermore, rare isoforms (JM-c and JM-d) were differentially expressed in the two tumor types, with both expressing JM-c and only medulloblastoma expressing the JM-d variant. Neither of these rare isoforms were detected in normal brain (86).

Ependymoma is the third most common brain tumors in children (88). Histological grading for these tumors is controversial, hence there is much interest in identifying molecular targets that can be used as prognostic markers (93). Next generation sequencing of an ependymoma tumor found previously unknown mutations for this cancer, including an *ERRB4* variant that has been detected in lung adenocarcinoma (93). A study of a large cohort of pediatric ependymoma patients found that co-expression of ErbB4 with ErbB2 occurred in over 70% of cases and was associated with higher proliferative index and poorer patient survival (88). The JM-a/CYT-1 and JM-a/CYT-2 isoforms were most highly expressed, with very little JM-b detected (88).

High grade gliomas (HGGs) are one of the most common forms of cancer affecting the central nervous system (94). HGG commonly targets adults, but as many as 12% of children diagnosed with primary CNS tumors suffer from HGG (95). HGG typically originates in non-neuronal cells, including glial cells, oligodendrocytes, and ependymal cells, in the brain and spinal cord (94). As expected, given the extensive role it plays during brain development, studies have identified ErbB4 as a potential target in HGG (10, 96, 97).

The most studied HGG is GBM, the most common and aggressive primary brain tumor in adults (98). Reports on the expression of ErbB4 in this disease are contradictory, with some reports suggesting that expression levels may influence whether ErbB4 plays an oncogenic or tumor suppressor role (99). ErbB4 mRNA and protein is expressed in the GBM cell lines SKMG‐3, SF767, U87, T98, and LN-229, as well as in primary GBM cells (10, 100–103). However, quantitative RT-PCR analysis of GBM patient samples found that whilst *EGFR* mRNA was overexpressed, *ERBB4* mRNA remained at a level similar to normal brain (80). Moreover, a study by Andersson and colleagues showed that ErbB4 mRNA and protein expression was highest in low-grade gliomas, compared with higher grade tumors, suggesting that ErbB4 may act as a tumor suppressor (100). However, the results of this study were variable, and some GBM samples expressed comparatively high levels of ErbB4, both at the mRNA and protein level (100). Also, this study did not use isoform-specific antibodies and qPCR probes, and therefore, did not assess expression of the different ErbB4 isoforms (100). Comparison of ErbB4 isoform-specific expression in low versus high-grade glioma may be more pertinent. An isoform-specific RT-qPCR analysis of patient-derived GBM cell lines found JM-a/CYT-2 to be the predominantly expressed ErbB4 isoform, which is different from the usual JM-b predominance in neural tissue (10, 73). As previously stated, the JM-a/CYT-2 isoform has the capacity to autophosphorylate without ligand binding and thereby increase cell proliferation and decrease apoptosis (52, 68, 69). It is, therefore, possible that the JM-a/CYT-2 isoform performs these oncogenic functions in GBM, contributing to the aggressiveness of this disease. A recent report using patient-derived GBM samples and xenograft models of GBM also found significant associations between ErbB4 mRNA levels and tumorigenicity, proliferation, angiogenesis, and therapeutic response (10). Patients with high levels of ErbB4 activation had significantly shorter survival than those with little or no ErbB4 activation (10). These findings support the potential role of ErbB4 as a prognostic and therapeutic target in GBM.

Immunohistochemical studies of GBM patient samples have also attempted to relate ErbB family expression patterns to survival outcomes, with conflicting results. As outlined above, the 2004 study by Andersson and colleagues found variable expression of ErbB4 protein in GBM samples (100). Another study examined expression profiles of the ErbB family members in a panel of nine GBM patient samples (104). Whilst ErbB4 was expressed in the vast majority of GBM samples, expression levels were lower in GBM samples than in controls. Moreover, in GBM patient samples, ErbB4 was mainly detected in neuronal-like elements and occasionally in hypertrophic astrocytes (104).

Other studies report more significant ErbB4 expression levels in GBM. Using immunohistochemistry, Bodey and colleagues reported elevated ErbB4 expression in pediatric HGG samples compared with normal brain controls, with ‘strong immunoreactivity’ in GBM samples (97). Nabika and colleagues examined expression profiles of ErbB1–4 and the Cyclin-Dependent Kinases p21 and p27 in high grade (III and IV) gliomas (105). High ErbB1/EGFR and ErbB4 expression, along with low p27 expression, was associated with poor patient outcome (105). In addition, high ErbB4 expression was an independent indicator for poor survival (105). Further studies are required to understand these conflicting results and to clearly define the role that ErbB4 plays in GBM.

In addition to isoform-specific expression levels, mutations may influence ErbB4 function in GBM. Circulating tumor cell clusters in GBM were first identified in 2018 and sequencing analysis identified a structural variant of the *ERBB4* gene within these clusters (106). Moreover, targeted next generation sequencing identified *ERBB4* mutations in a cohort of 228 primary GBM patients (107). Although these mutations are of unknown significance, in this cohort they were found to contribute to overall survival (107). Therefore, the authors speculated that, based on other data supporting a role for ERBB4 in GBM progression, these may be inactivating mutations (10, 107).

Molecular interactions can also affect ErbB4 signalling in GBM. The WWOX tumor suppressor protein can interact with full-length ErbB4 or with the ICD only, with distinct results (108). WWOX interaction with full-length ErbB4 stabilizes the ErbB4 receptor in the cellular membrane (108). In contrast, when interacting with the ICD only, WWOX prevents the ICD from reaching the nucleus (108). In 2019, Chen and colleagues reported a novel regulatory pathway involving ErbB4 and circular RNA, which is upregulated in glioma (GM) cells (109). The circular RNA, circ_0074026 was highly expressed in HGG samples and correlated with ERBB4 mRNA expression (109). This circular RNA targets miRNA-1304, which appears to modulate ERBB4 expression and possibly contribute to glioma progression (109).

Diffuse midline glioma (DMG), otherwise known as diffuse intrinsic pontine glioma (DIPG), is considered the pediatric counterpart of GBM (110). DMG tumors are rarely surgically removed or biopsied, and consequently, they have not been molecularly characterized to the same level as many other primary brain tumors, such as GBM (111, 112). However, FISH analysis of genetic expression profiles comparing DIPG samples with non-DIPG pediatric GBM found recurrent focal gains for *ERBB4* (96).

As previously discussed, ErbB4 can heterodimerize with the other ErbB family members, with each dimer having unique signalling properties. For example, since EGFR is dysregulated in approximately 70% of GBMs, the EGFR/ErbB4 heterodimer would almost certainly be present in tumors and may even be the dominant signalling moiety (113). Likewise, the presence of ErbB2 and ErbB3 has the capacity to alter the function of ErbB4 in tumors. Thus, additional studies are required to determine how the co-expression of ErbB family members may influence the function of ErbB4 in patients.

In summary, research is limited regarding individual ErbB4 isoform expression in brain tumors. However, analysis of ErbB4 isoform differential expression in medulloblastoma and ependymoma has highlighted the association between JM-a isoform expression and higher proliferative indices, translating to poorer survival rates (87, 88). Comparison of isoform-specific expression in low- versus high-grade glioma has not been reported, however the JM-a isoform appears to be the dominant isoform in GBM, and this may contribute to tumor aggressiveness (10). Future mechanistic studies are required to clarify the role of the JM-a isoform in GBM progression.

## ErbB4 and possible therapeutic implications in high grade glioma

Currently there are no small molecule inhibitors that specifically inhibit ErbB4. There are, however, inhibitors that block the activity of ErbB4 in conjunction with other ErbB family members. We have demonstrated that the pan-ErbB inhibitor dacomitinib could inhibit the growth of GBM and pediatric medulloblastoma in orthotopic xenograft models (114). Similar results have been obtained with a second pan-ErbB inhibitor, NT113, in models of adult GBM (115). However, it remains unknown whether the inhibition of ErbB4 enhances or impedes the efficacy of these drugs. Another possible approach might be the repurposing of ibrutinib, which was designed to target Bruton’s tyrosine kinase. A recent report demonstrated that it inhibited ErbB4 at low concentrations and showed some preferential efficacy towards high-expressing ErbB4 cell lines (116).

The effective use of agents that inhibit ErB4 in brain cancer will require research that identifies pro-tumorigenic forms of ErbB4; targeting tumor suppressor forms of ErbB4 with drugs could obviously be detrimental to patients. As previously discussed, mutations of ErbB4 have been described in a range of brain cancers and the presence of such activating mutations could identify patients who may benefit from ErbB4 therapeutics. We recently demonstrated that the presence of highly phosphorylated ErbB4 in GBM, independent of phosphorylated EGFR, was linked to shorter patient survival compared to its absence (10). Additionally, in xenograft models, increased ErbB4 activation in GBM cells was associated with increased tumorigenicity, proliferation, and angiogenesis, as well as reduced sensitivity to anti-EGFR treatments (10). This strongly suggests that in GBM, activation of ErbB4 is pro-tumorigenic and thus phosphorylated ErbB4 could be a second biomarker for predicting response to ErbB4 targeting drugs. The mechanisms by which ErbB4 activation increases high-grade glioma aggressiveness are not yet understood. Indeed, there may be other viable targets within this pathway.

No specific ErbB4 inhibitors are currently available. Furthermore, the implications of inhibiting ErbB4 function in the brain, particularly the developing child brain, must be considered. As previously established, ErbB4 has important roles in normal brain physiology and vital roles in brain development (25). Thus, it is reasonable to speculate that negative side effects could occur in patients administered an ErbB4 inhibitor. It may be possible to limit side effects by developing isoform-specific inhibitors that target only the oncogenic ErbB4 isoform JM-a/CYT-2. Using approaches such as CRISPR in patient-derived cell lines will allow us to determine the role of the different isoforms, such as JM-a/CYT-2, in the growth and survival of brain cancer cells. These are the critical studies required before moving ErbB4 therapeutic forward into the clinic. Developing inhibitors for specific ErbB family members has proven a difficult task and may take a significant amount of time and resources to achieve. It is therefore imperative to elucidate ErbB4 signalling mechanisms in HGG and establish the impact of this pathway on disease progression and therapeutic resistance before developing such inhibitors.

We have shown that the interaction between c-MET and EGFR leads to the transactivation of both receptors in GBM (117). It is likely that ErbB4 can also be activated by other receptors expressed in brain cancers. Indeed, as recently extensively reviewed, many G-protein-coupled receptors can activate members of the ErbB family, including ErbB4 (118). Therefore, when determining the role of ErbB4 in brain cancers, other mechanisms of activation apart from ligand engagement must be considered. Identifying the presence of activated ErbB4, rather than the simple presence of ErbB4 protein, may be the most important approach to determining if ErbB4 is a driver in patient tumors (10).

Lessons should be learnt from the development and trial of EGFR-specific inhibitors in GBM treatment. While promising in pre-clinical models, EGFR inhibitors have had inconsistent results in clinical trials (119). This is believed to be due to the highly plastic and heterogenous nature of high-grade gliomas (119). Ultimately, a single target may not be enough to treat these cancers. Perhaps a multi-faceted approach with both ErbB4 inhibitors and EGFR inhibitors could prove more consistently effective against high-grade gliomas.

## Future directions

The ‘uniqueness’ of ErbB4 in the ErbB receptor family, epitomized by its isoform variations, tissue- and disease state-specific expression, vast array of activating ligands (and yet, a seemingly selective subset of downstream targets), and large number of tyrosine phosphorylation sites makes it an attractive potential target to treat diseases such as GBM. However, these unique characteristics, in conjunction with the lack of inhibitors and other high-quality reagents for detecting its activation, and its ability to homo- and heterodimerize with other members of the ErbB receptor family, also make it inherently complicated to study.

Clearly, more mechanistic studies are required to further elucidate the role that ErbB4 may play in the progression of GBM. Current therapies do not adequately address the diffuse nature of this tumor or the plasticity of the signalling pathways that underlie its progression. Therefore, further insights into the role of ErbB4 in this disease are warranted.

## Author contributions

The draft of this manuscript was prepared equally by J-LP and NA. All authors edited, reviewed, and approved the final version of the manuscript.

## Funding

The research work related to the review in the author’s lab is funded by the Robert Connor Dawes Foundation in association with the Pirate Ship Foundation. At the time of writing, the first author of this review was receiving the support of the Australian Government Research Training Program Scholarship at The University of Western Australia.

## Acknowledgments

The authors thank Leah Cannon, PhD, for editing this manuscript.

## Conflict of interest

The authors declare that the research was conducted in the absence of any commercial or financial relationships that could be construed as a potential conflict of interest.

## Publisher’s note

All claims expressed in this article are solely those of the authors and do not necessarily represent those of their affiliated organizations, or those of the publisher, the editors and the reviewers. Any product that may be evaluated in this article, or claim that may be made by its manufacturer, is not guaranteed or endorsed by the publisher.
